# Supplementary material for: Anticancer Potential of Natural Chalcones: In Vitro and In Vivo Evidence
Source: Int J Mol Sci. 2023 Jun 19;24(12):10354. doi: 10.3390/ijms241210354 (PMC10299153; doi:10.3390/ijms241210354)
Supplement: Supplementary file 1 [file ijms-24-10354-s001.zip › ijms-2439404-supplementary.pdf]

Table S1. Antiprolierative and anticancer effects of xanthohumol and butein based on *in vitro*/*in vivo* studies.

Chalcones	Type of cancer	Mechanism of action/target or/and downstream effector	References
Xanthohumol 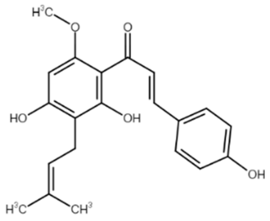	Breast	In vitro: inhibition of proliferation, migration, colony and sphere formation<br>↓ mitochondrial function, ↓ ROS production, ↑ population of sub G0/G1 cells, induction of apoptosis, ↓ expression of Bcl-2, MMP-9, MDR1, EGFR, STAT3, ↑ expression of DR4 and DR5, sensitization of MCF-7/ADR cells, PI3K, HER2 and Ser/Thr kinase receptors binding activity<br>In vivo: antiangiogenic effect, ↓ expression of factor VIII, phospho- IκBα, IL-1b, Ki-67, Notch 1, activity of NFκB,<br>↓ activation of Notch signaling pathway	[24-26,28,29,31, 32,345]
	Liver	In vitro: induction of apoptosis, inhibition of cell proliferation, migration and clonogenicity<br>↓ Notch1, HES-1, Notch signaling pathway, NF-κB<br>↑ caspase-3 activation, p53<br>In vivo: reduction the number of liver preneoplastic lesions	[90-93,95]
	Pancreas	In vitro: suppression of cancer cells growth, inhibition of colony formation, tube formation and angiogenesis, induction of apoptosis,<br>↓ Notch signaling, NF-κB signaling, phospho-STAT3, VEGF, IL-8, expression of cyclin D1, survivin and Bcl-xL genes<br>In vivo: reduction of volume and weight of tumors, inhibition of NF-κB signaling pathway	[127-130]
	Lungs	In vitro: inhibition of cell proliferation, colony formation, migration and invasion, DNA fragmentation/damage, cell cycle arrest in G1, S and G2/M phase, induction of mitochondrial pathway of apoptosis, inhibition of TOPO I<br>↓ cyclin D1, Ras/Raf/MEK/Erk pathway, phospho-Erk1/2, p90RSK, pCREB, Fra1, phospho-TOPK, phospho-Akt, phospho-histone H3, ABCB1, ABCC1, ABCC2, ABCC3, MMP-2, MMP-9, VEGF, TGF-β, vimentin, N-cadherin, Snail	[170-175,177-180]

		↑ p53, p21, p27, PUMA, Bax, Bak, cytochrome c release, activation of caspase -3, -8, -9, TIMP-1, E-cadherin, α-catenin<br>In vivo: reduction of tumor weight and volume<br>↓ Ki-67, cyclin D1, Fra1<br>↑ PUMA	
	Prostate	In vitro: inhibition of cancer cells proliferation, migration and invasion, apoptotic (extrinsic and intrinsic) and autophagic cell death, S phase cell cycle arrest, mitochondrial damage, induction of oxidative stress<br>↓ mitochondrial membrane potential, phospho-Akt, phospho-mTOR, NF-κB signaling, Bcl-2, Bcl-xL, survivin<br>↑ activation of caspase -3, -8, -9, PARP cleavage, cytochrome c release, Bid, Bax<br>In vivo: reduction of progression and tumor growth	[256-262,264]
	Skin	In vitro: inhibition of proliferation and colony formation and migratory activity<br>↓ phospho-p38, phopsho-JNK, phospho-Erk<br>In vivo: inhibition of proliferation, decrease of number and size of metastases<br>↓ MIA, Ki-67 positive cells	[330,331,333]
Butein<br>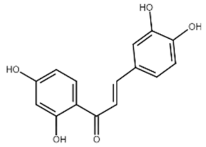	Breast	In vitro: inhibition of cell proliferation and clonogenic growth, cell cycle arrest, aromatase inhibition<br>↓ PMA-induced expression of COX-2, CXCR4, CXCL12-induced migration and invasion, phosphorylation of Erk, Bcl-2, ER-α expression, ↑ p38 activity, caspase-3 and PARP cleavage, phospho-STAT3<br>In vivo: inhibition of the growth of ERα+ breast cancer cells, ↓ ROS, inhibition of Akt phosphorylation	[36-43]
	Liver	In vitro: cell proliferation suppression, G2/M cell cycle arrest, apoptosis induction, DNA damage response, inhibition of invasion and migration<br>↓ MMP-2, MMP-7, MMP-9, uPA, Ras, Rho A, ROCK1, MEKK3, Erk1/2, JNK1/2, phospho-c-Jun, Akt/mTOR/p70S6K signaling pathway<br>↑ phospho- Chk1, phospho-Chk2, ROS generation, DR5 receptor, phospho-p38<br>In vivo: inhibition of tumor growth, ↓ Aurora B kinase, STAT3 signaling pathway	[96,97,100-103]
	Pancreas	In vitro: ↓ expression of CXCR4, NF-κB pathway	[37]

	Oral cavity	In vitro: induction of apoptosis, inhibition of migration and invasion<br>↓ phospho-NF-κB/p65, COX-2, MMP-9, survivin	[146]
	Lungs	In vitro: inhibition of cell proliferation, colony formation and migration, reduction of cell viability, G0/G1 and G2/M cell cycle arrest, DNA fragmentation, induction of mitochondrial and ER pathway of apoptosis<br>↓ cyclin D, cyclin B1, cdc25C, cdc2, MMP, Bcl-2, SOD2, GSH, phospho-EGFR, MET kinase activity, phospho-ERB3, phospho-Akt, phospho-Erk1/2, PD-L1, STAT1<br>↑ Bax, PUMA, activity of caspase -3, -8, -9, ROS generation, phospho-PERK, phospho-eIFα, ATF4, CHOP, IRE1α, XBP1, cleavage of PARP, phospoho-p38<br>In vivo: inhibition of tumor growth, reduction of volume and weight of tumor, induction of ER stress, ROS generation<br>↓ Bcl-2, PD-L1<br>↑ CHOP, Bax	[43,184,185,187,188,190]
	Prostate	In vitro: inhibition of cell viability and proliferation, migration, invasion and angiogenesis<br>↓ cyclin D1, cyclin D2, cyclin E, Cdk2, Cdk4, Cdk6, Bcl-2, phospho-PI3K, phospho-Akt, IκB, NF-κB, VEGF, MMP-9<br>↑ p21, p27, activation of caspase -3, -8, -9, PARP cleavage, Bax<br>In vivo: inhibition of tumor growth<br>↓ Ki-67 positive cells, VEGF, CD31	[265,266]
	Bladder	In vitro: antimigratory and antiinvasive activity, inhibition of EMT<br>↓ phospho-Erk1/2, NF-κB signaling pathway, vimentin<br>↑ E-cadherin	[307]

Table S2. Antiproliferative and anticancer effects of cardamonin and licochalcones based on *in vitro*/*in vivo* studies.

Chalcones	Type of cancer	Mechanism of action/target or/and downstream effector	References
Cardamonin<br>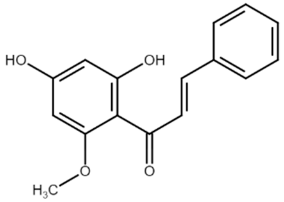	Breast	In vitro: inhibition of proliferation, EMT transition, adhesion, migration, angiogenesis, induction of cell cycle arrest, DNA fragmentation and apoptosis<br>↓ Bcl-2, N-cadherin, vimentin, β-catenin, Slug, Snail, HIF-1α, cyclin D1, c-Myc, VEGF, Cdk-4, phospho-Akt, phospho-GSK-3β, phosho-S6K1, phospho-mTOR<br>↑ Bax, cytochrome c, cleaved caspase -3, cleaved PARP, E-cadherin, ROS accumulation, PD-L1<br>In vivo: dose-dependent suppression of tumor volume, ↓ HIF-1α	[55,56,58-60]
	Liver	In vitro: induction of extrinsic and intrinsic apoptosis<br>↓ NF-κB pathway<br>↑ ROS generation<br>In vivo: suppression of tumor growth, inhibition of NF-κB pathway<br>↓ PCNA, Ki-67, Bcl-2, NF-κB/p65, Ikkβ<br>↑ Bax	[104,105]
	Lungs	In vitro: inhibition of cell proliferation, colony formation, migration and invasion, cell cycle arrest v G2/M phase, DNA fragmentation, nuclear condensation, induction of apoptosis, mitochondrial damage, modulation of tumor microenvironment and EMT<br>↓ cyclin D1, Cdk4, Bcl-2, N-cadherin, ZEB1, Snail, phospho-JNK, phospho-Akt, phospho-mTOR, S6K1<br>↑ Bax, activation of caspase -3, cleaved PARP, ROS generation, E-cadherin, PP2A<br>In vivo: reduction in volume and size of tumors, reduction of number of metastatic nodules<br>↓ phospho-Akt, phospho-mTOR, Ki-67, NF-κB signaling	[200-202,205,207,346]
	Prostate	In vitro: induction of cell death, DNA fragmentation, inhibition of migration and invasion<br>↓ NF-κB, STAT3, JAK2, Bcl-xL, Bcl-2, survivin, XIAP, VEGF, COX2, MMP-9, cyclin D1, cyclin E, Cdk2, Cdk4	[272,273]

		↑ activation of caspase -3, -8, -9, PARP cleavage	
Licochalcones 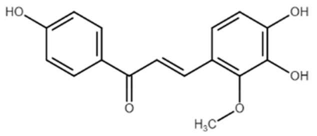 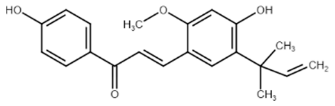	Breast	In vitro: inhibition of proliferation, invasion, migration and resistance, induction of DNA damage, autophagy and apoptosis, G2/M cell cycle arrest<br>↓ Bcl-2, cyclin D1, PI3K/Akt/mTOR pathway, PRMT6, MMP, AURKA, MDR-1, Sp-1, vimentin, phospho-Akt, phospho-JNK, phospho-p38, phospho-NF-κB, p62<br>↑ Bax, PARP cleavage, p53, cleaved caspase -3, -9, cytochrome c release, E-cadherin, Beclin-1, ATG5	[62,64-69,347]
	Liver	In vitro: inhibition of migration, invasion, cell proliferation, induction of extrinsic and intrinsic apoptosis, G2/M cell cycle arrest<br>↓ uPA mRNA, uPA, Akt/mTOR pathway, mRNA of survivin, cyclin B1, CdK1, Akt, p70S5K<br>↑ ROS generation, MAPK pathway, expression of DR4, DR5, mRNA of Wee1, p21, DR3, DR5, caspase -3, -8, -10, Fas, Bad, Bax, Bak, PUMA, FOXO signaling<br>In vivo: inhibition of HCC cell-mediated lung metastasis, prevention of (DEN)-induced DNA damage/fragmentation and antiapoptotic proteins expression	[106-113]
	Oral cavity	In vitro: induction of extrinsic and intrinsic apoptotic pathways, inhibition of migration<br>↓ Sp1, PI3K/Akt pathway, JAK/STAT pathway, survivin, cyclin D1, Bcl-2, Bcl-xL, matrin 3<br>↑ FasL, phospho-Erk, phospho-p38, p27, activation of caspase -3, -8, -9, PARP cleavage, DNA fragmentation, Bax, Bad, ROS production	[137-145]
	Esophagus	In vitro: induction of endoplasmic reticulum stress and apoptosis, ROS generation, cell cycle arrest in G1 and G2/M phase<br>↓ cyclin B1, cyclin D1, JAK2 kinase activity, phospho-STAT3, cdc2, Mcl-1, Bcl-2, mitochondrial cytochrome c<br>↑ p21, p27, MAPK pathway, phospho-JNK, phospho-c-Jun, phosho-p38, GRP78, CHOP, DR4, DR5, tBid, Bax, cytosolic cytochrome c, Apaf-1, cleaved PARP	[150-152]
	Lungs	In vitro: suppression of tumor cells proliferation, DNA damage, G1 and G2/M cell cycle arrest, mitochondrial and ER-mediated apoptotic pathway, induction of autophagy, ROS generation, inhibition of migration and invasion<br>↓ MMP, cyclin B1, cyclin D1, cdc2/Cdk1, Cdk2, Cdk4, cdc25C, MDM2, Bcl-2, Bcl-xL, Mcl-1, Nrf2, γ-GCSc, c-IAP1, c-IAP2, XIAP, c-FLIP$_L$, PI3K/Akt/mTOR pathway, phospho-	[208-211,213-215,219,222,225,226]

		JNK1, Wnt/β-catenin signaling, phospho-EGFR, phospho-c-Met, PD-L1, phospho-4EBP1, MMP-1, MMP-3, Sp1<br>↑ Bax, Bad, Bid, cytochrome c release, Apaf-1, activation of caspase -3, -7, PARP cleavage, phospho-ATF4, phospho-PERK, eIF2, CHOP, BiP, ATG1, ATG3, ATG6, ATG16, LC3-I/II, miR-133-3p, phospho-p38, p21, p27, DR4, DR5<br>In vivo: reduction of tumor volume and size<br>↓ PI3K/Akt signaling, Ki-67, c-Met, phospho-EGFR, survivin	
	Prostate	In vitro: suppression of tumor cells growth, G1 and G2/M cell cycle arrest, induction of apoptosis, inhibition of migration<br>↓ cyclin B1, cdc2, Rb	[274,275]
	Bladder	In vitro: inhibition of cell proliferation, induction of mitochondrial dysfunction, ER-stress and apoptosis cell death, ROS generation, G1 and G2/M cell cycle arrest<br>↑ activation of caspase -3, -9, Bax, Bim Apaf-1, cleavage of PARP<br>↓ GSH, cyclin A, cyclin B1, Wee1, Cdk1, Cdk2, cdc25A, cdc25B, NF-κB/p65, MMP-9, Bcl-2, Bcl-w, Bcl-xL<br>In vivo: reduction of tumor size and weight, anti-migratory, anti-invasive and anti-adhesive activity	[311-316]
	Skin	In vitro: inhibition of cell proliferation, induction of apoptosis, inhibition of migration and invasion, ROS generation<br>↓ MITF, mTOR signaling pathway, Bcl-2, mitochondrial membrane potential, p62, MM-2, MMP-9<br>↑ activation of caspase -3, -9, Bax, LC3-I/II, Beclin-1, ATG1, ATG5<br>In vivo: inhibition of tumor growth	[335,336]

Table S3. Antiproliferative and anticancer effects of isoliquiritigenin, flavokawains and garcinol based on *in vitro*/*in vivo* studies.

Chalcones	Type of cancer	Mechanism of action/target or/and downstream effector	References
Isoliquiritigenin	Breast	In vitro: antiproliferative, antiangiogenic, antimetastatic, antiinvasive activity, dual ER (estrogenic) agonist activity<br>↓ cell viability, BrdU incorporation, metabolism of arachidonic acid, PI3K/Akt signaling pathway, cell cycle regulating proteins, β-catenin transcription activity, miR-374a, expression of Bcl-2, total and phosphorylated mTOR, VEGF-receptor, COX-2, RANKL/OPG ratio<br>↑ autophagy-mediated apoptosis, miR-200c, expression of PTEN, Bax, activation of caspase-3 and PARP, HIF-1α degradation,<br>In vivo: ↓ c-Jun, ↑ miR-200c	[45-52]
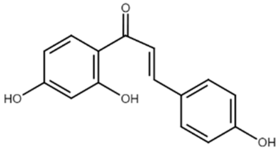	Oral cavity	In vitro: downregulation of DNA repair mechanisms, induction od DNA damage and apoptosis, G2/M cell cycle arrest, inhibition of colony formation and invasion<br>↓ expression of ATM, ALDH1, CD44, ABCG2, GRP78	[147,148]
	Lungs	In vitro: induction of extrinsic and intrinsic apoptotic cell death and necrosis, G0/G1 and G2/M cell cycle arrest, DNA fragmentation, mitochondrial damage, inhibition of EMT, invasion and migration<br>↓ Bcl-2, MDM2, phospho-EGFR, phospho-Akt, phospho-Erk1/2, cyclin D, PI3K/Akt signaling, mTOR, p70, Wnt/β-catenin pathway, β-catenin nuclear translocation, N-cadherin, vimentin, phospho-FAK, phospho-cortactin<br>↑ Fas, mFasL, sFasL, p53, p21, Bax, Bim, cleaved caspase -3, -7, -8, -9, cleaved PARP, E-cadherin<br>In vivo: inhibition of tumor growth and size, reduction of number and size of metastatic lesions<br>↓ phospho-FAK, phospho-cortactin, Src signaling pathway	[191-195,197,198,300,348-350]
	Prostate	In vitro: reduction of cell growth, G1 and G2/M cell cycle arrest, inhibition of migration, invasion, adhesion<br>↓ phospho-cdc2, MMP-9, uPA, TIMP-1, VEGF, integrin-α2, ICAM, VCAM, phospho-Akt, phospho-JNK	[267,268,270,271]

		↑ cdc25C, p38 MAPK signaling, Erk signaling,<br>In vivo: reduction of tumor growth, volume and size, induction of apoptosis<br>↓ Cdk1, cyclin B1<br>↑ activation of caspase -3	
	Kidneys	In vitro: reduction of cell viability, inhibition of proliferation, migration and invasion, induction of apoptosis and autophagy, ROS generation<br>↓ Bcl-2, Bcl-xL, STAT3 activity, cyclin D1, cyclin D2, PI3K/Akt/mTOR signaling pathway<br>↑ activation of caspase -3, -7, -9, PARP cleavage, Bax, cytochrome c release<br>In vivo: reduction the number of metastatic nodules	[298-300]
	Bladder	In vitro: induction of apoptosis, chromatin condensation, nuclear fragmentation, G1 phase cell cycle arrest, inhibition of migration, ROS generation<br>↓ Bcl-2, mitochondrial membrane potential<br>↑ Bim, Bax, Apaf-1, caspase -3, -9, cdc2	[308-310]
	Skin	In vitro: inhibition of proliferation, migration and EMT, ROS generation, induction of apoptosis,<br>↓ GLUT1/4, hexokinase 2, pyruvate kinase M2, LDH, mitochondrial membrane potential, HIF-1α, miRNA27a, vimentin, miR-301b, miRNA-431-5p<br>↑ E-cadherin	[337-341]
Flavokawains<br>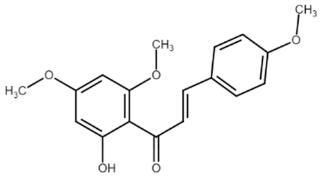	Breast	In vitro: inhibition of cell proliferation, motility and invasiveness, colony formation, induction of apoptosis, cell cycle arrest in G1 and G2/M phase, immunomodulatory and anti-inflammatory effect<br>↓ MMP, angiogenic activity, VEGF, CAM1, phospho-cdc2, phospho-Cdc25C, Myt1, Wee1, MPM-2, HER2, phospho-Akt, Bcl-2, Bcl-xL, survinin, XIAP<br>↑ activation of caspase -8, -9, p27, Bax, Bim, cytochrome c, histone H1, cleaved PARP<br>In vivo: ↓ tumor weight and volume<br>↑ T cells, IFN-γ, IL-2 in serum, CD3, CD4, CD8, NK1.1	[71-73]
	Lungs	In vitro: inhibition of tumor cell growth and proliferation, G2/M cell cycle arrest, ROS production, induction of apoptotic and autophagic cell death<br>↓ Bcl-xL, XIAP, surivin, PI3K/Akt/mTOR signaling, P-gp<br>↑ Bax, cytochrome c release, activation of caspase -3, -7, -9, cleavage of PARP, LC3-I/II, ATG4B, ATG7, JNK and p38 pathway	[227,228,230,231]
	Prostate	In vitro: inhibition of cell proliferation, G2/M cell cycle arrest, induction od apoptosis<br>↓ Skp2, Cdh1, survivin, tubulin polymerisation, glutamine metabolism, SGH	[276-280]

		↑ p27, ROS production<br>In vivo: reduction of tumor growth<br>↓ NANOG, OCT4, CD44, c-Myc, survivin, XIAP, Skp2<br>↑ Bax, Bim, PUMA, DR5, p21, p27, caspase activation	
	Bladder	In vitro: suppression of cell growth, intrinsic pathway of apoptosis, G1 and G2/M cell cycle arrest<br>↓ Bcl-xL, mitochondrial membrane potential, Cdk2, Cdk1<br>↑ activation of caspase -3, -9, Bax, cytochrome c release<br>In vivo: induction of apoptosis, increase of survival<br>↓ Ki-67 positive cells, PRMT5	[318-320,323]
	Skin	In vitro: antiproliferative activity, induction of autophagy and apoptosis, AVO formation<br>↓ Bcl-2, Akt/mTOR pathway, BRAF<br>↑ activation of caspase -3, PARP cleavage, LC3-I/II, p62<br>In vivo: reduction of tumor weight and volume	[334]
Garcinol<br>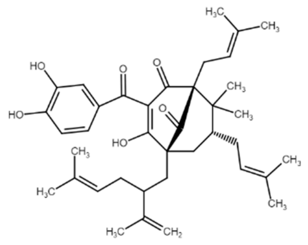	Breast	In vitro: inhibition of proliferation, G0/G1 cell cycle arrest, induction of apoptosis, EMT and metastasis, inhibition of acetyltransferase<br>↓ vimentin, acH3, NF-κB/ac-p65, NF-κB nuclear translocation, cyclin D1, Bcl-2, Bcl-xL, ZEB1, ZEB2, cytosolic and nuclear β-catenin<br>↑ E-cadherin, miR-200, Iet-7 miRNAs	[75-77]
	Pancreas	In vitro: inhibition of cell proliferation and migration, induction of apoptosis<br>↓ NF-κB, MMP-9, VEGF, IL-1, STAT3	[131,132]
	Lungs	In vitro: G1 and S phase cell cycle arrest, reduction and viability, proliferation, sefl-renewal, multidrug resistance, EMT and DNA reparation<br>↓ cyclin D1, cyclin D3, cyclin E, Cdk2, Cdk4, Cdk6, p38 MAPK pathway, c-FLIP, OCT4, BMI1, SOX2, NANOG, Notch1, ABCG2, c-Myc, phospho-STAT3, survivin, phospho-JAK1, phospho-JAK2, MAPK, Wnt/β-catenin signaling pathway, LRP6, β-catenin, Dvl2, Axin2, ALDH1A1, N-cadherin, vimentin<br>↑ p21, p27, DR5, DDIT3, E-cadherin<br>In vivo: reduction of size and weight of tumor<br>↓ Ki-67 positive cells, ALDH1A1<br>↑ DDIT3	[232-234,236-238]
	Prostate	In vitro: inhibition of tumor cell proliferation, induction of apoptosis and autophagy, DNA fragmentation	[281,282]

		↓ mitochondrial membrane potential, Bcl-2, Erk1/2, NF-κB<br>↑ activation of caspase -3, -9, PARP cleavage, Bax LC3-I/II, phospho-mTOR, GSK-3β, PI3K, Akt, PDK1	

References

345 Gupta, K.K.; Sharma, K.K.; Chandra, H.; Panwar, H.; Bhardwaj, N.; Altwaijry, N.A.; Alsfouk, A.A.; Dlamini, Z.; Afzal, O.; Altamimi, A.S.A.; et al. The integrative bioinformatics approaches to predict the xanthohumol as anti-breast cancer molecule: Targeting cancer cells signaling PI3K and AKT kinase pathway. *Front. Oncol.* **2022**, *12*, 950835. https://doi.org/10.3389/fonc.2022.950835.

346 Makhija, P.; Handral, H.K.; Mahadevan, G.; Kathuria, H.; Sethi, G.; Grobben, B. Black cardamom (*Amomum subulatum Roxb*.) fruit extracts exhibit apoptotic activity against lung cancer cells. *J. Ethnopharmacol.* **2022**, *287*, 114953. https://doi.org/10.1016/j.jep.2021.114953.

347 Wahyuni, W.; Diantini, A.; Ghozali, M.; Subarnas, A.; Julaeha, E.; Amalia, R.; Sahidin, I. Cytotoxic and Antimigration Activity of Etlingera alba (A.D.) Poulsen Rhizome. *Adv. Pharmacol. Pharm. Sci.* **2021**, *2021*, 6597402. https://doi.org/10.1155/2021/6597402.

348 Cui, Y.; Wu, Y.; Wang, C.; Wang, Z.; Li, Y.; Jiang, Z.; Zhao, W.; Pan, Z. Isoliquiritigenin inhibits non-small cell lung cancer progression via m6A/IGF2BP3-dependent TWIST1 mRNA stabilization. *Phytomed. Int. J. Phytother. Phytopharm.* **2022**, *104*, 154299. https://doi.org/10.1016/j.phymed.2022.154299.

349 Han, C.; Kawata, M.; Hamada, Y.; Kondo, T.; Wada, J.; Asano, K.; Makabe, H.; Igarashi, K.; Kuzumaki, N.; Narita, M.; et al. Analyses of the possible anti-tumor effect of yokukansan. *J. Nat. Med.* **2019**, *73*, 468–479. https://doi.org/10.1007/s11418-019-01283-x.

350 Zhou, Y.; Ho, W.S. Combination of liquiritin, isoliquiritin and isoliquirigenin induce apoptotic cell death through upregulating p53 and p21 in the A549 non-small cell lung cancer cells. *Oncol. Rep.* **2014**, *31*, 298–304. https://doi.org/10.3892/or.2013.2849.
